# A Preaching Match: The Sunday Fund

**Published:** 1887-06-18

**Authors:** 


### A PREACHING MATCH:

### THE SUNDAY FUND.

"THUS custom doth make *dotards* of us all," says Carlyle, stealing from Shakespeare a setting for his own diamond. The particular light-points contributed by the truculent sage are "custom" and "dotards," all the rest being the property of the poet. "Dotards" is a good rousing word, well calculated to set the teeth on edge and to excite a man to the knocking-down point. If a respectably dressed citizen meets another in the street, and gently hints that half-a-crown would be an accommodation at the moment, the probabilities are that the stranger will pass on with a bow of the stiffest and a smile hardly polite. But if the half-crown seeker stands squarely in front of his fellow-being, and says, "Sir, you're a dotard, or at least a confounded fool," he will certainly receive some kind of attention, even if it be only that of the nearest policeman.

Custom makes men dotards, says the prophet of Craigenputtock, and he included women. What is a dotard? Primarily a person of feeble mind, of weak will, of defective intelligence; an incapable, inefficient, poor, spiritless, helpless creature, the condition of the man being generally the result of advancing age. We imagine ourselves to be addressing men and women, and picture ourselves standing well in front of them in the character of a sandwich-man, with Carlyle's aphorism painted in big black letters on a white ground on each of the immense boards between which we are sandwiched. Let not the profane reader smile. The writer of an article has some purpose to serve when he consents to become a sandwich-man.

### CUSTOM MAKES MEN AND WOMEN DOTARDS.

Stand still and look at the large letters on the board. A man gives half-a-crown for a new necktie, and half-a-crown as his annual contribution to all the sick and dying poor of the metropolis. A mother buys her small baby a new doll for a shilling, and offers exactly a shilling to all the hospitals which provide for the sick children, and sick men and women too, of the poorer classes of this vast London. Now where is the sense of fitness in such a judgment of the case? The curious thing is that both the man and woman call themselves Christians, that is to say, they claim to belong to a society whose only *raison d'être* is that its members are striving after divine perfection. "Be ye therefore perfect, even as your Father which is in heaven is perfect," says the preacher from the pulpit. Everybody listens with the fullest approbation, and admits that the text is in exact accordance with his own particular opinions. "Whatsoever ye would that men should do unto you, do ye even so unto them," sounds from the lectern. Nobody questions the propriety of the utterance; nobody smiles, at any rate so long as the church walls enclose him, whatever he may do in the City, or at his club on Monday.

Well, but have we not, after all, a little degree of intelligence, or at least some sense of the humorous? What if our favourite dog or horse were to be lost: should we give a sum counted in shillings for their recovery? Is it really true, then, that the men and women who go to church in garments and jewellery which at a moderate estimate may often be valued at not less than fifty pounds, will think they have done well when they have put a guinea into the plate for the seventy metropolitan charities? If it be, they must be dotards; they cannot but be stupid, imbecile, incapable, worthless, and miserable creatures. It would be breaking a fly on the wheel to invoke a Dante to denounce a doom upon them. What worse doom can they have than to be conscious that no really thoughtful person would willingly spend five minutes in their company; that in the world to come they will probably not be found worthy of either reward or punishment, but simply of contempt?

The late Dean Ramsay, in his *Reminiscences of Scottish Life and Character*, gives some amusing sketches of the "Preaching Matches" which used to take place at communion-time in the outlying districts of Scotland many years ago. In those simple times grave Doctors of Divinity did not hesitate to put forth all their powers in order to carry off the palm. We may smile and think our tastes in these times much superior to those of ancient Presbyterian—not to speak of Round-headed divines. But, at any rate, there need be no hesitation among the clergy and other ministers of religion, in trying who shall be the most successful pleader on behalf of the hospitals and medical charities. The theme is eminently Christian and human; the object altogether such as no man can call in question.

Perhaps one reason why custom has so stereotyped the contributions of the pew, is that the same custom has stereotyped the platitudes of the pulpit. Sir Edmund Currie, speaking at a small hospital meeting at Blackheath, the other evening, said pathetically, "If it had been announced that I should come to this meeting with my face blackened, and give a performance with the bones, the place would have been full." Not a doubt of it; and whilst we do not advise the clergy to preach with blackened faces, much less to attempt any demonstration with "the bones," we do urge them, with painful urgency, to make some effort to rid themselves of ineffective commonplaces, and to persuade or shame their audiences into something like a juster understanding of the subject. Forty thousand pounds from the whole of London on Hospital Sunday is perfectly ridiculous. Many years ago a country vicar, who had formerly been an Indian chaplain, had a collection for an Indian Famine Fund. The amount collected was about six pounds. The vicar, when he heard of the sum, got into a most Christian rage. He placarded the little town during the week, and announced another collection for next Sunday. The Sunday came, and the sermon. Nobody was very comfortable during its delivery. The parish had no squire, or other large proprietor, but the doctor, the lawyer, the bank agent, the prosperous draper, and all the smaller fry of shopkeepers heard some plain speaking for once in their lives. At the close of the sermon the preacher said, "You all know my family,"—he had eleven children, all young—"and most of you know the amount of my income. I shall give ten pounds. We shall see what you give." The second collection was not six pounds, but ninety.

Will not the clergy in this Jubilee Year take some means of thoroughly rousing up their hearers to a right view of the subject, and endeavour to provoke in them a permanent enthusiasm for those good deeds which are the very soul and essence of the faith which they preach?

